# Gadolinium Enhanced MR Coronary Vessel Wall Imaging at 3.0 Tesla

**DOI:** 10.4061/2010/856418

**Published:** 2010-10-11

**Authors:** Sebastian Kelle, Kelly Schlendorf, Glenn A. Hirsch, Gary Gerstenblith, Eckart Fleck, Robert G. Weiss, Matthias Stuber

**Affiliations:** ^1^Department of Medicine/Cardiology, Deutsches Herzzentrum Berlin, Augustenburger Platz 1, 13353 Berlin, Germany; ^2^Division of Magnetic Resonance Research, Department of Radiology, Johns Hopkins University, School of Medicine, 601 North Caroline Street, Baltimore, MD 21287, USA; ^3^Division of Cardiology, Department of Medicine, Johns Hopkins University, Baltimore, MD 21205-2196, USA; ^4^Department of Electrical and Computer Engineering, Johns Hopkins University, Baltimore, MD 21205-2196, USA

## Abstract

*Purpose*. We evaluated the influence of the time between low-dose gadolinium (Gd) contrast administration and coronary vessel wall enhancement (LGE) detected by 3T magnetic resonance imaging (MRI) in healthy subjects and patients with coronary artery disease (CAD). *Materials and Methods.* Four healthy subjects (4 men, mean age 29 ± 3 years and eleven CAD patients (6 women, mean age 61 ± 10 years) were studied on a commercial 3.0 Tesla (T) whole-body MR imaging system (Achieva 3.0 T; Philips, Best, The Netherlands). T1-weighted inversion-recovery coronary magnetic resonance imaging (MRI) was repeated up to 75 minutes after administration of low-dose Gadolinium (Gd) (0.1 mmol/kg Gd-DTPA). *Results.* LGE was seen in none of the healthy subjects, however in all of the CAD patients. In CAD patients, fifty-six of 62 (90.3%) segments showed LGE of the coronary artery vessel wall at time-interval 1 after contrast. At time-interval 2, 34 of 42 (81.0%) and at time-interval 3, 29 of 39 evaluable segments (74.4%) were enhanced. *Conclusion*. In this work, we demonstrate LGE of the coronary artery vessel wall using 3.0 T MRI after a single, low-dose Gd contrast injection in CAD patients but not in healthy subjects. In the majority of the evaluated coronary segments in CAD patients, LGE of the coronary vessel wall was already detectable 30–45 minutes after administration of the contrast agent.

## 1. Introduction

Although invasive X-ray coronary angiography (XCA) is the current clinical standard for visualizing the coronary artery lumen,it does not directly image the vessel wall and often underestimates the extent of underlying atherosclerotic burden. These disadvantages,as well as the requirement for intracoronary contrast administration, limit its value for early disease detection, characterization of plaque components [1], and for following the impact of disease-modifying therapeutic interventions. Magnetic resonance imaging (MRI) provides excellent soft tissue characterization and allows the noninvasive evaluation of atherosclerotic plaques in animal models [2] and patients [3, 4]. 

Despite the constant motion, small caliber and deep location of the coronary arteries within the chest, the *in vivo* coronary vessel wall in proximal and mid coronary artery segments can often be well visualized by 1.5 T MRI [5, 6]. Simultaneously, late gadolinium-enhanced (LGE) MRI of the vascular wall is a very promising method for evaluating carotid and aortic plaque, inflammatory vasculopathies, and for identifying some plaque components, including the fibrous cap [7–10].

Initial coronary artery vessel wall LGE studies demonstrated the potential utility of LGE-MRI at 1.5 T for coronary plaque visualization and characterization using a T1-weighted MRI technique with an inherent high contrast between enhancing and nonenhancing tissue [1, 11–13]. However, in all these early studies, a double or triple dose of Gadolinium (Gd) was injected, the time course of enhancement was not well characterized despite a wide range of image acquisition-times (60 to 180 minutes post contrast administration) [1, 11–13], and investigations were limited to 1.5 T magnetic field strength only. 

In this study, we examined coronary artery vessel wall LGE in healthy subjects and patients with established and stable CAD by using 3.0 T MRI after a single, low-dose contrast injection. We also investigated the effect of time from contrast administration to the MRI scan on coronary vessel wall LGE.

## 2. Materials and Methods

### 2.1. Study Population and Design

For imaging of healthy subjects, the study was conducted in accordance with the standards of the Charité institutional ethics committee. For MRI of CAD patients, the study protocol was approved by the Johns Hopkins Institutional Review Board and all CAD patients gave written informed consent prior to participation. Four healthy subjects (4 men, age 29 ± 3) and eleven patients (5 men, 6 women, age 44–76 years, mean 61 ± 10 years) were prospectively enrolled. Healthy subjects were defined as those without a history of CAD and the absence of traditional CAD risk factors other than male gender. All CAD subjects had a clinical history of CAD, defined by previous revascularization including percutaneous coronary intervention, coronary artery bypass grafting, or prior myocardial infarction. Patient demographics are summarized in Table 1.

### 2.2. Magnetic Resonance Coronary Artery Lumen and Wall Imaging

All participants were examined in the supine position using a commercial 3.0 T whole-body MR imaging system (Achieva 3.0 T; Philips, Best, The Netherlands) equipped with a Quasar Dual gradient system (80 mT/m, 200 T/m/s slew rate). A six-element cardiac phased-array coil was used for signal reception. Cardiac synchronization was performed using a vector electrocardiogram [14]. All subjects underwent a standardized MR examination consisting of the following steps.

For localization of the heart in the three standard planes (axial, coronal, and sagittal), a rapid segmented k-space gradient echo imaging sequence (multistack, multislice survey scan, TR/TE/flip angle = 3.8 ms/1.8 ms/20°) was used. This scan was also used for localization of the respiratory navigator. Next, an axial mid-ventricular cine-segmented k-space gradient echo imaging sequence with 50 cine frames was performed to visually determine the individual rest period of the coronaries in diastole. Timing of the coronary MRI acquisition within the cardiac cycle (trigger delay) was then adapted to each patient's individual coronary artery rest period. For gating and subsequent correction of diaphragmatic motion during free breathing, a navigator (gating window: 5 mm) was placed at the dome of the right hemidiaphragm.

A bolus of 0.1 mmol/kg bodyweight Gd-DTPA (low/single-dose) (Magnevist, Berlex Laboratories, Montville, New Jersey, USA) at an injection rate of 2 mL/s, followed by a flush of 20 mL of saline solution at the same rate, was then administered intravenously. Magnetic resonance coronary angiography (MRCA) of the right or left coronary artery system after contrast was performed with a previously described navigator-gated free-breathing and fat-suppressed, cardiac-triggered T2-prepared segmented k-space gradient echo imaging sequence [15]. The acquired voxel size was 0.70 × 1.01 × 3.00 mm^3^, reconstructed to 0.70 × 0.70 × 1.50 mm^3^.

The subsequent LGE scans of the coronary vessel wall were performed with a navigator-gated free breathing and cardiac-triggered T1-weighted inversion-recovery and fat-suppressed 3D black-blood segmented k-space gradient echo imaging sequence. Parameters of the sequence were. TR/TE/flip angle = 4.7 ms/1.6 ms/20°. Spatial resolution of the sequence was 0.99 × 1.04 × 2.00 mm^3^ and reconstructed to 0.53 × 0.53 × 1.00 mm^3^. The inversion time (TI) of the inversion recovery sequence used to null blood was typically >300 ms. Parallel imaging (SENSE (sensitivity encoding), reduction factor 2) was used, as it allows for shorter scan times. Scans of the right and left coronary artery system were performed in the same orientation as the earlier acquired T2prep-images, to ensure adequate coregistration. The duration of a typical LGE scan was between 2:14 and 4:46 minutes depending on the heart rate and breathing pattern of the patient. This scan protocol for LGE of the coronaries was repeated as often as possible from 30 minutes to a maximum of 75 minutes after contrast administration. In the four healthy subjects and two of the CAD patients, inversion recovery measurements were performed prior to contrast administration.

### 2.3. MR Image Analysis

The unprocessed MRI images were used to assess the coronary artery segments on a segment-by-segment basis as visible or nonvisible on T2prep-images by consensus of two interpreters blinded to the XCA results, clinical data, and time postadministration of Gd. For assessment of coronary artery segments, a quantitative coronary analysis tool (Soap-Bubble Tool) [16] was used. Coronary artery segments were classified using a modified 7-segment-model, according to the recommendations from the AHA [17]. In brief, this included the left main and proximal and mid segments of the left anterior descending artery (LAD), left circumflex artery (LCX), and right coronary artery (RCA). Coronary artery segments with stents or coronary artery bypass grafts (CABG) artifacts were excluded because of potential artifacts.

In every acquired scan, all coronary artery segments with LGE were assigned to the corresponding coronary segments seen on T2prep-images and the number of enhanced segments as well as the degree of enhancement was recorded. LGE of the coronary vessel wall was rated on a 3-point-scale: 0 = no enhancement, 1 = mild to moderate enhancement, and 2 = strong enhancement. In addition, the presence or absence of aortic wall enhancement of the ascending and descending aorta was assessed and the degree of enhancement using the same 3-point scale was documented.

The data were then grouped into three time-intervals based on how long after contrast administration the images were obtained. Time-interval 1 was 30–45 minutes after contrast, time-interval 2 was 46–60 minutes after contrast, and time-interval 3 was 61–75 minutes postcontrast. 

Contrast between coronary vessel wall LGE and blood was quantified. Regions of interest were drawn at the coronary vessel wall and in the blood pool and signal intensities (SI) were recorded. Contrast was determined by dividing the SI of coronary wall LGE by SI of blood.

### 2.4. Statistical Analysis

Statistical analysis was performed using SPSS for Windows (release 12.0.1; SPSS, Chicago, Ill, USA). All continuous parameters were shown as mean ± one standard deviation (SD). The Chi-square-test was used to compare the prevalences of LGE of the coronary vessel wall among the three time-intervals. Logistic regression analysis was used to test for trend for the prevalence of LGE among the three time-intervals. For comparison of inter-observer variability related to coronary vessel wall enhancement on a per-segment basis, kappa values were calculated. To detect differences in the mean coronary and aortic LGE scores among the three time-intervals, the Kruskal-Wallis test was performed. Wilcoxon signed-rank test was used to compare the visual assigned score of the degree of LGE among the different time-intervals for the coronary as well as the aortic vessel wall and to compare aortic wall LGE and coronary vessel wall LGE at every time-interval. To account for multiple comparisons of LGE among the three time-intervals, the *p*-value was corrected using the Bonferroni method. A *p*-value of <0.05 was considered statistically significant.

## 3. Results

**Figure 1:**
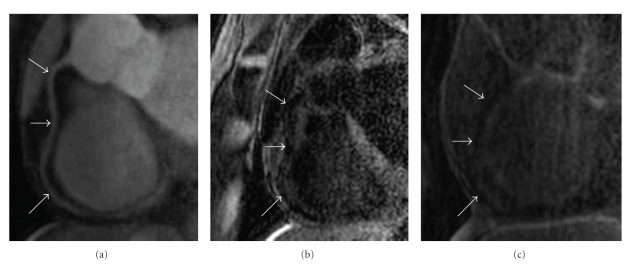
(a) Formatted coronary magnetic resonance angiography (MRCA) of the right coronary artery (RCA) indicated by white arrows. (b) No enhancement of the coronary artery vessel wall can be observed on the precontrast (b) and 30 minutes postcontrast administration (c) acquired inversion-recovery coronary vessel wall scans of the RCA.

Three patients were excluded from the analysis because of nondiagnostic contrast-enhanced images: one for ECG triggering difficulties, one for inadequate nulling of blood signal, and another who terminated the study prematurely because of discomfort while in the MRI scanner. MR coronary artery imaging was successfully performed in the four healthy subjects and the remaining eight CAD patients. Thirty-one inversion recovery measurements were performed (one in every healthy subject and 3.4 ± 2.3 per CAD patient). LGE was seen in none of the healthy subjects. All 27 measurements demonstrated LGE in at least one of the evaluated coronary artery segments. In the two patients in whom inversion recovery scans were performed prior to contrast administration, twelve segments were evaluable and no segment demonstrated LGE prior to contrast administration. However, all of these segments showed enhancement post contrast (Figure 1).

### 3.1. MR Angiographic Parameters

There was substantial agreement between both observers in the determination of coronary artery vessel wall enhancement on a per-segment basis (*κ* = 0.74; *p* < 0.001). At time-interval 1, data of 7 patients; at time-interval 2, data of 6 patients, and at time-interval 3, data of 5 patients were available for analysis. The prevalence of LGE of the coronary vessel wall was not significantly different when comparing the three time-intervals. Fifty-six of 62 (90.3%) visible segments by T2Prep showed coronary vessel wall LGE at time-interval 1. At time-interval 2, 34 of 42 (81.0%) segments showed enhancement and at time-interval 3, 29 of 39 segments (74.4%) were enhanced (Figure 3). A comparison of the prevalence of LGE between time-interval 1 and 2 as well as between time-interval 2 and 3 revealed no statistically significant difference (*p* = 0.17 and *p* = 0.48). However, logistic regression analysis demonstrated a significant trend to a lower prevalence of LGE from the earliest, time-interval 1, to the latest time-interval, time-interval 3, (*p* = 0.03).

In a subgroup of 3 patients who had undergone coronary X-ray angiography within 3 months of MRI, the prevalence (%) of LGE in coronary artery segments with and without wall irregularities at coronary X-ray angiography in CAD patients over time is shown in Figure 4. The highest prevalence of LGE in coronary segments with CAD was found at time-interval 1.

The mean coronary vessel wall LGE score was similar for the first two intervals (time-interval 1: 1.29 ± 0.64 and time-interval 2: 1.28 ± 0.80), although the enhancement at time-interval 3 (0.97 ± 0.71) was significantly lower than that at time-interval 1 (*p* = 0.027). 

The average aortic wall enhancement was similar for the three time-intervals (time-interval 1: 1.50 ± 0.80; time-interval 2: 1.81 ± 0.40; time-interval 3: 1.81 ± 0.54) and did not differ significantly (*p* = 0.293) as a function of time post-contrast. A significantly higher degree of enhancement of the aortic wall after 46–60 minutes (*p* = 0.005) and after 61–75 minutes (*p* = 0.02) was found when compared to coronary vessel wall enhancement at the same time-interval (Figure 5). 

The mean coronary vessel wall to blood signal contrast was similar at all three time-intervals. The contrast ratio at time-interval 1 (1.62 ± 0.49) was not significantly different from that at time-interval 2 (1.57 ± 0.42) (*p* = 0.63), and that at time-interval 2 was not significantly different from that of time-interval 3 (1.58 ± 0.35) (*p* = 0.69). Similarly, no difference between time-interval 1 and time-interval 3 (*p* = 0.88) was found.

## 4. Discussion

This study of healthy subjects and patients with stable CAD demonstrates several important findings: LGE of the coronary artery vessel wall was commonly detected at 3.0 T after single administration of low-dose Gd in patients with X-ray-defined CAD but not in healthy subjects. Secondly, in CAD patients, a substantial enhancement in the majority of the evaluated segments was already present between 30 and 45 minutes after contrast administration.

One theoretical advantage of 3.0 T over 1.5 T includes an increase of the signal-to-noise ratio and therefore the ability to achieve a higher spatial resolution and/or accelerated image acquisition. These potential advantages make 3.0 T well suited for coronary artery imaging [18–20]. 

To our knowledge, there are only a few published studies [1, 11, 13] or case reports [12] describing LGE of the coronary artery vessel wall at 1.5 T. For cardiovascular magnetic resonance (CMR) [21] and especially LGE of myocardial scar tissue, one of the major advantages of 3.0 T versus 1.5 T seems to be the possibility of a reduced contrast agent dose [22] as well as the potential for higher spatial and temporal resolution [23]. Previous reports on LGE of the coronary vessel wall at 1.5 T reported the use of contrast dosages from 0.2 mmol/kg/body weight [11–13] to 0.3 mmol/kg/body weight [1], which is two to three times higher than the dosage used in our study (0.1 mmol/kg/body weight of Gd-containing contrast) in patients with a normal glomerular filtration rate (81.3 ± 22.6 mL*min^−1∗^(1.73 m^2^)^−1^). The ability to use a lower contrast agent dose is promising, especially in light of recently published reports identifying a possible link between a scleroderma-like disorder, nephrogenic systemic fibrosis (NSF), and exposure to Gd-containing contrast agents in patients with end-stage renal disease [24]. Independent of the potential risks of NSF, single dose of Gd reduces the costs per exam.

We obtained measures of coronary LGE at 3.0 T. We used a slightly higher spatial resolution for LGE of the coronary artery vessel wall (0.99 × 1.04 × 2.00 mm^3^) at 3.0 T compared to previously published 1.5 T studies from Maintz et al. (1.00 × 1.00 × 3.00 mm^3^) [1] and Yeon et al. (1.25 × 1.25 × 3.00 mm^3^) [13].

In previously published studies at 1.5 T, the time of LGE measurements of the coronary vessel wall varied widely and ranged from 30 minutes after contrast [11, 12] to 60 minutes [13] and even 3 hours after Gd administration [1]. No characterization of the time course of the enhancement process has been reported. In our study group, a significant trend for a higher prevalence of LGE (*p* = 0.03) at the earliest time-interval was demonstrated, with substantial enhancement of the coronary artery vessel wall already present at 30–45 minutes after contrast administration (Figure 6). In addition, quantitative measurements of mean coronary vessel wall contrast enhancement were similar at all three observed time-intervals. In our study, the prevalence of LGE was relatively high when compared to earlier reports from studies conducted at 1.5 T. This may be attributable to differences between the patient cohorts and/or in the time interval between Gd injection and imaging. These are some of the earliest coronary wall LGE data following contrast administration and the limited available data suggest that the percent of enhanced-CAD segments is higher shortly after contrast administration than at later times (Figure 4). The optimal timing of MRI after contrast administration to precisely characterize coronary atherosclerosis may well require additional studies directly comparing the time course of LGE to the histopathologic extent of local coronary atherosclerosis.

Yeon et al. compared LGE-measurements of the coronary vessel wall with coronary plaque detection by multislice-computed tomography (MSCT) and >20% luminal narrowing by XCA in patients with coronary risk factors [13]. They reported that among their patients generally demonstrated coronary artery wall enhancement and 66% of the evaluable segments showed LGE. In our study, all of whom had established and severe CAD, 82.2% of all coronary segments demonstrated LGE. In the same study by Yeon et al., the prevalence of LGE increased with severity of coronary artery disease by MSCT along the spectrum from no plaque to noncalcified plaque to calcified plaque. The authors stated that LGE contrast uptake may be associated with an increased distribution volume (as with fibrosis and neovascularization) in the altered vessel wall or with increased vascular permeability (as may occur with inflammation). In our study, no evidence of LGE before contrast was found but related data were only obtained in all of the healthy and only two of the patients.

**Figure 2:**
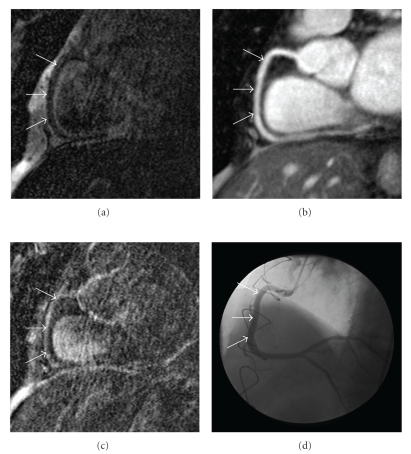
(a) No enhancement of the coronary artery vessel wall can be observed on the pre-contrast inversion-recovery coronary vessel wall scans of the right coronary artery (RCA). (b) Formatted coronary magnetic resonance angiography (MRCA) in the same orientation demonstrating no high-grade stenosis. (d) Corresponding invasive catheterization of the RCA confirming MRCA findings. (c) Contrast uptake of coronary artery vessel wall of the RCA is demonstrated 45 minutes after contrast administration. The RCA vessel lumen or vessel wall is indicated by white arrows.

**Figure 3:**
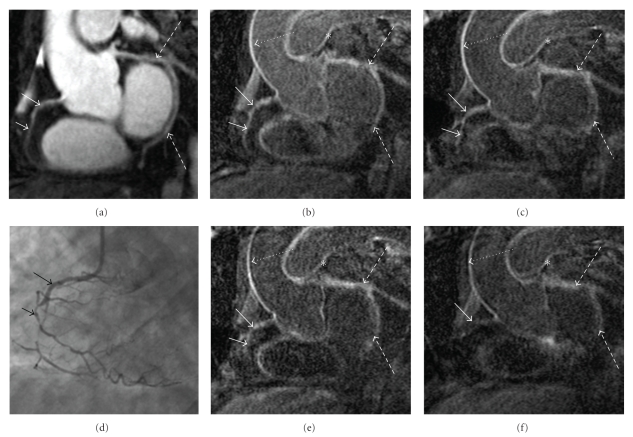
(a) MR-coronary angiography of the RCA (white arrows) showing intraluminal signal loss at medial and distal RCA as demonstrated by coronary angiography (d) (black arrows). Reformatted inversion recovery MR images: (b) 33 minutes; (c) age range 50 minutes; (e) 68 minutes, and (f) 77 minutes post contrast administration demonstrate strong enhancement of the coronary vessel wall at the earliest time-points and signal loss over the time-intervals (white arrows). Note strong enhancement of the vessel wall of the aortic arch (dashed arrows) in contrast to the loss of enhancement of the RCA (solid arrows) and LCX (interrupted arrows) over time. There is also enhancement of the pulmonary artery (asterisk).

**Figure 4:**
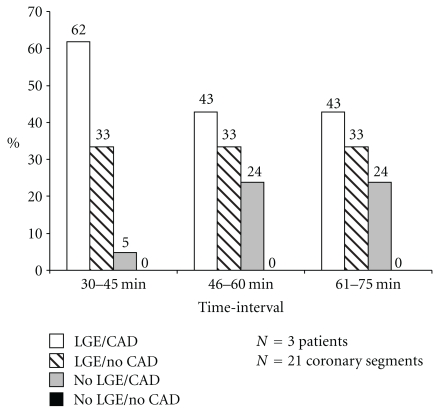
Prevalence (%) of LGE in coronary artery segments with and without wall irregularities at coronary X-ray angiography in CAD patients over time.

**Figure 5:**
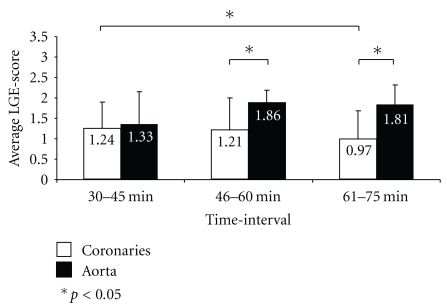
Bar graph demonstrating the average-LGE score over time in coronary artery segments and aorta. *indicates statistical significance (*p* < 0.05).

**Figure 6:**
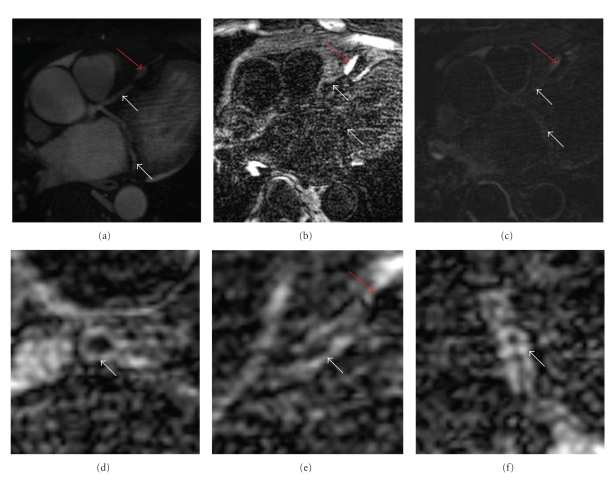
(a) Formatted coronary magnetic resonance angiography (MRCA) of the LAD and LCX demonstrating no high-grade stenosis, but a stent artifact in the mid LAD which is indicated by a red arrow. (b) No enhancement of the coronary artery vessel wall can be observed on the pre-contrast inversion-recovery coronary vessel wall scans. (c) Contrast uptake of coronary artery vessel wall of the LAD and LCX is demonstrated 30 minutes after contrast administration. The vessel wall of the left main (d); LAD (e), and LCX (f) is indicated by white arrows.

A high degree of aortic wall enhancement was observed for all 3 time-intervals and the visually assessed LGE-score after 46–60 and 61–75 minutes was greater than that of the coronary vessel wall at the same time. The greater aortic vessel wall thickness than that of the coronary arteries may contribute to this observation. Also the pulmonary artery trunk showed LGE in healthy subjects and CAD patients, as seen in Figure 2. A potential reason for this might be different wall composition between the coronary arteries and the pulmonary trunk and should be evaluated in further studies. 

In a clinical setting, our findings may have important implications for the design of MRI protocols that include post-contrast imaging of the coronary artery vessel wall. On one hand, coronary artery vessel wall enhancement can be studied early after the administration of the contrast agent which abbreviates overall scanning time. On the other hand, coronary LGE can be added to the end of a more prolonged comprehensive cardiac MRI exam up until 60 minutes after contrast injection, albeit at the expense of a slightly reduced conspicuity of the contrast between enhanced and non-enhanced-coronary vessel wall.

## 5. Limitations

The number of subjects in this study is relatively small, but consistent with other initial studies of a new technology implemented at higher magnetic field strength. No direct comparison was made to subjects examined at 1.5 T or to different doses of Gd. Furthermore, scans were not performed at every time-interval in every patient, due to the inability of many CAD patients to tolerate long scan times. However, the findings here better define the time course of LGE after Gd contrast administration and may help to guide protocol definition of future studies related to LGE of the coronary arteries. 

No comparison with histopathologic data was performed, as coronary pathologic data are only available at autopsy. In addition, we did not compare LGE of the coronary vessel wall with intravascular ultrasound, the current in vivo gold standard for coronary plaque assessment. 

Unfortunately only 3 patients had recent X-ray coronary angiograms to verify the presence of atherosclerosis in the enhanced segments. It would be important in future studies to include more patients and directly compare invasive X-ray or IVUS with pre- and post-contrast LGE of the coronary vessel wall to better characterize the type and extent of atherosclerosis in LGE regions (as opposed to non-LGE regions). The effect of different time intervals on LGE of the coronary vessel wall compared to patients with cardiac risk factors but no established clinical disease needs to be addressed in future studies. In addition, non-contrast imaging of the coronary vessel wall prior to the acquisition of contrast-enhanced coronary vessel wall imaging should be considered.

The LGE sequence was initially developed for visualization of myocardial scar and is routinely used in clinical practice between 10 and 20 minutes post-contrast administration [25]. However, image quality at this relatively early time point may be limited by contrast agent which has not completely washed out of the blood-pool. For this reason, LGE coronary imaging was performed 30 minutes and beyond in the present study. To optimize image quality in every scan, a look-locker-sequence [26] for an accurate definition of the inversion time for blood-signal nulling may be useful as well. However, 30 minutes after the administration of a single dose of Gd, the T1 changes are expected to be minimal.

Finally, due to the use of SENSE, a quantitative signal-to-noise (SNR) and contrast-to-noise (CNR) evaluation of the coronary artery vessel wall was not performed.

## 6. Conclusions

In healthy subjects, no coronary LGE was seen. In patients with stable CAD, the combination of 3 T MRI and a single, low dose Gd injection enabled the detection of coronary LGE over a relatively long period of time. Most coronary segments showed LGE with the largest proportion of the evaluated coronary segments already enhancing 30–45 minutes after Gd-administration. This new information will likely have important implications for the design of MRI protocols that include post-contrast imaging of the coronary artery vessel wall.

##  Conflict of Interests

The author(s) declare that they have no competing interests.

## Figures and Tables

**Table 1:** Patient characteristics. BMI = body mass index; CAD = coronary artery disease; PCI = percutaneous coronary intervention; CABG = coronary artery bypass graft; LVEF = left ventricular ejection fraction (derived from echocardiography); GFR = glomerular filtration rate. Values are expressed as mean ± one standard deviation.

Patient characteristics	
sex, F/M	6/5
age, y	61 ± 10
age range	44–76
BMI, kg/m^2^	27.1 ± 4.2

Medical history information, *n* (%)	

hypertension	9 (82)
diabetes mellitus	4 (36)
hyperlipoproteinemia	9 (82)
history of smoking	4 (36)
previous PCI	7 (64)
previous CABG	4 (36)
prior myocardial infarction	6 (55)
LVEF (%)	50.5 ± 9.0
GFR (mL*min^−1∗^(1.73 m^2^)^−1^)	81.3 ± 22.6

Medication	

beta-blocker	6 (55)
statins	10 (91)
ACE-inhibitors	5 (45)

## References

[1] Maintz D, Ozgun M, Hoffmeier A (2006). Selective coronary artery plaque visualization and differentiation by contrast-enhanced inversion prepared MRI. *European Heart Journal*.

[2] Weinreb DB, Aguinaldo JGS, Feig JE, Fisher EA, Fayad ZA (2007). Non-invasive MRI of mouse models of atherosclerosis. *NMR in Biomedicine*.

[3] Kramer CM, Cerilli LA, Hagspiel K, DiMaria JM, Epstein FH, Kern JA (2004). Magnetic resonance imaging identifies the fibrous cap in atherosclerotic abdominal aortic aneurysm. *Circulation*.

[4] Saam T, Hatsukami TS, Takaya N (2007). The vulnerable, or high-risk, atherosclerotic plaque: noninvasive MR imaging for characterization and assessment. *Radiology*.

[5] Botnar RM, Stuber M, Kissinger KV, Kim WY, Spuentrup E, Manning WJ (2000). Noninvasive coronary vessel wall and plaque imaging with magnetic resonance imaging. *Circulation*.

[6] Kim WY, Stuber M, Börnert P, Kissinger KV, Manning WJ, Botnar RM (2002). Three-dimensional black-blood cardiac magnetic resonance coronary vessel wall imaging detects positive arterial remodeling in patients with nonsignificant coronary artery disease. *Circulation*.

[7] Desai MY, Stone JH, Foo TKF, Hellmann DB, Lima JAC, Bluemke DA (2005). Delayed contrast-enhanced MRI of the aortic wall in Takayasu’s arteritis: initial experience. *American Journal of Roentgenology*.

[8] Yuan C, Kerwin WS, Ferguson MS (2002). Contrast-enhanced high resolution MRI for atherosclerotic carotid artery tissue characterization. *Journal of Magnetic Resonance Imaging*.

[9] Cai J, Hatsukami TS, Ferguson MS (2005). In vivo quantitative measurement of intact fibrous cap and lipid-rich necrotic core size in atherosclerotic carotid plaque: comparison of high-resolution, contrast-enhanced magnetic resonance imaging and histology. *Circulation*.

[10] Wasserman BA, Smith WI, Trout III HH, Cannon RO, Balaban RS, Arai AE (2002). Carotid artery atherosclerosis: in vivo morphologic characterization with gadolinium-enhanced double-oblique MR imaging—initial results. *Radiology*.

[11] Ibrahim T, Dirschinger J, Schachoff S (2006). Darstellung der Koronargefaesswand mittels kontrastunterstuetzter Magnet-Resonanz-Tomographie bei Patienten mit koronarer Herzerkrankung. *Clinical Research in Cardiology*.

[12] Wessely R, Botnar RM, Vorpahl M, Schwaiger M, Schömig A, Ibrahim T (2007). Images in cardiovascular medicine. Subacute thrombotic occlusion and spontaneous recanalization of the right coronary artery after percutaneous coronary intervention for ST-elevation myocardial infarction visualized by coronary angiography and cardiac magnetic resonance imaging. *Circulation*.

[13] Yeon SB, Sabir A, Clouse M (2007). Delayed-enhancement cardiovascular magnetic resonance coronary artery wall imaging. Comparison with multislice computed tomography and quantitative coronary angiography. *Journal of the American College of Cardiology*.

[14] Fischer SE, Wickline SA, Lorenz CH (1999). Novel real-time R-wave detection algorithm based on the vectorcardiogram for accurate gated magnetic resonance acquisitions. *Magnetic Resonance in Medicine*.

[15] Nezafat R, Stuber M, Ouwerkerk R, Gharib AM, Desai MY, Pettigrew RI (2006). B1-insensitive T2 preparation for improved coronary magnetic resonance angiography at 3 T. *Magnetic Resonance in Medicine*.

[16] Etienne A, Botnar RM, Van Muiswinkel AMC, Boesiger P, Manning WJ, Stuber M (2002). "Soap-Bubble" visualization and quantitative analysis of 3D coronary magnetic resonance angiograms. *Magnetic Resonance in Medicine*.

[17] Scanlon PJ, Faxon DP, Audet A-M (1999). ACC/AHA Guidelines for Coronary Angiography. A report of the American College of Cardiology/American Heart Association Task Force on Practice Guidelines (Committee on Coronary Angiography). Developed in collaboration with the Society for Cardiac Angiography and Interventions. *Journal of the American College of Cardiology*.

[18] Bi X, Deshpande V, Simonetti O, Laub G, Li D (2005). Three-dimensional breathhold SSFP coronary MRA: a comparison between 1.5T and 3.0T. *Journal of Magnetic Resonance Imaging*.

[19] Bi X, Carr JC, Li D (2007). Whole-heart coronary magnetic resonance angiography at 3 Tesla in 5 minutes with slow infusion of Gd-BOPTA, a high-relaxivity clinical contrast agent. *Magnetic Resonance in Medicine*.

[20] Stuber M, Börnert P, Spuentrup E, Botnar RM, Manning WJ (2002). Selective three-dimensional visualization of the coronary arterial lumen using arterial spin tagging. *Magnetic Resonance in Medicine*.

[21] Pintaske J, Martirosian P, Graf H (2006). Relaxivity of gadopentetate dimeglumine (Magnevist), gadobutrol (Gadovist), and gadobenate dimeglumine (MultiHance) in human blood plasma at 0.2, 1.5, and 3 Tesla. *Investigative Radiology*.

[22] Kelle S, Kokocinski T, Thouet T (2005). 1.5 vs. 3.0 Tesla—reduction of contrast agent dosage for scar-imaging possible?. *European Heart Journal *.

[23] Klumpp B, Fenchel M, Hoevelborn T (2006). Assessment of myocardial viability using delayed enhancement magnetic resonance imaging at 3.0 Tesla. *Investigative Radiology*.

[24] Marckmann P, Skov L, Rossen K (2006). Nephrogenic systemic fibrosis: suspected causative role of gadodiamide used for contrast-enhanced magnetic resonance imaging. *Journal of the American Society of Nephrology*.

[25] Kim RJ, Shah DJ, Judd RM (2003). How we perform delayed enhancement imaging. *Journal of Cardiovascular Magnetic Resonance*.

[26] Look DC, Locker DR (1970). Time saving in measurement of NMR and EPR relaxation times. *Review of Scientific Instruments*.

